# Beneficial Alterations of Intestinal Microbiota in Chronic Cholecystitis Patients Treated With NOTES Gallbladder-Preserving Surgery

**DOI:** 10.1155/2024/9327118

**Published:** 2024-11-07

**Authors:** Lixin Deng, Xinzhi Lv, Taotao Wang, Xishun Huang, Qingrong Huang, Xianli Li, Chunhong Wen, Li Chen, Huidi Chen, Mingqing Zhang

**Affiliations:** ^1^Department of Gastroenterology, The 909th Hospital, School of Medicine, Xiamen University, Zhangzhou, Fujian, China; ^2^Department of Health Medicine, The 909th Hospital, School of Medicine, Xiamen University, Zhangzhou, Fujian, China

**Keywords:** 16S rDNA, benign gallbladder disease, chronic cholecystitis, intestinal microbiota, natural orifice transluminal endoscopic surgery, short-chain fatty acids

## Abstract

**Objective:** NOTES gallbladder-preserving surgery (N-GPS) has been heralded as a new paradigm shift in minimally invasive surgery for chronic cholecystitis patients. The objective of this research was to evaluate the impact of N-GPS on the intestinal microbiota of patients.

**Methods:** The study selected patients with benign gallbladder disease (BG group) within 1 week preoperative (BG_DPR stage) and followed up over 1 year postoperative (BG_YPO stage) and selected healthy controls (HC group) whose sex, age, and BMI index matched with patients at BG_YPO stage, too. Accordingly, stool samples from healthy controls and two stages of patients with benign gallbladder disease were collected; among them, the selected samples were sent for 16S rDNA sequencing with Illumina MiSeq platform, and then, the combined samples were sent for short-chain fatty acid (SCFA) analysis with GC-MS platform.

**Results:** The result of alpha diversity of Shannon index showed that the difference among the two stages of BG group and HC group wasn't statistically significant, while the result of beta diversity based on the weighted UniFrac distance suggested that the structure of intestinal microbiota of BG group at YPO stage was closer to HC group. LEfSe analysis suggested that BG_YPO stage enriched genus, such as *Enterocloster* and *Hungatella_A_128155*, which improved bile acid metabolism. Compared with BG_DPR stage, BG_YPO stage and HC group enriched *Faecalibacterium* and *Roseburia*, but depleted *Streptococcus*, while fecal SCFA concentrations increased.

**Conclusion:** Patients with benign gallbladder disease and chronic cholecystitis after N-GPS treatment for over 1 year improved gut microbial community structure. With the improving bile acid metabolism, SCFA-producing bacteria increased and pathobionts decreased, which helped the intestinal microbiota structure of BG group at YPO stage restore and close to HC group.

**Trial Registration:** Chinese Clinical Trial Registry identifier: ChiCTR1900028267.

## 1. Background

Gallstones and gallbladder polyps (GPs) are major benign gallbladder diseases. In 2020, a meta-analysis literature based on a cross-sectional study showed that the overall prevalence of gallstones in Mainland Chinese has reached 11.0% [1]. Eighty percent of acute cholecystitis are caused by gallbladder stones; if the acute infection is not treated in time, it will cause serious complications [2]. GPs are found in more than 4% of adult abdominal ultrasonography [3]. Certain GPs can induce cholecystitis, too. Furthermore, GPs over 1 cm were considered as a risk of gallbladder cancer. Thus far, laparoscopic cholecystectomy (LC) has become the gold standard treatment for benign gallbladder diseases [4]. However, the lack of gallbladder function after cholecystectomy may lead to the development of a variety of short- and long-term complications and adverse effects, including common bile duct stones, duodenal-gastric reflux, diarrhea, abdominal distension, dyspepsia, and even an increased incidence of gastrointestinal tumors [5].

Natural orifice translumenal endoscopic surgery (NOTES) is a technique that uses transvisceral access to perform surgical procedures entirely through a natural orifice and has been heralded as a new paradigm shift in minimally invasive surgery (Supporting Information 1: Figure S1) [6]. With the advancement of technology, the feasibility, safety, and effectiveness of NOTES technology have been confirmed through animal experimental research and have entered the clinical application stage. Common incision-related complications such as wound infections, incisional hernias, postoperative pain, aesthetic disdain, and adhesions could be minimized or eliminated by NOTES [7]. NOTES gallbladder-preserving surgery (N-GPS) is the application of NOTES technology for removing gallstones and GPs with preserving the gallbladder, offering a potential alternative to LC for benign gallbladder diseases. In addition to minimizing common incision-related complications, it can maintain the integrity of the body's digestive system and the constancy of the internal environment.

The microbial ecosystem of the gastrointestinal tract is characterized by many microbial species that balance their lives using mutualistic strategies. The eubiosis/dysbiosis condition of the gut microbiota strongly influences our health and disease status [8]. The pathogenesis of cholesterol gallstones is still not clear, though studies suggest that gut microbiota dysbiosis plays an important role in their formation [9, 10]. Cholelithiasis is a complex disease caused by an imbalance of cholesterol metabolism, including intestinal absorption, liver synthesis, bile cholesterol output, and bile acid conversion. Genetic factors, environmental factors, and behavioral habits also play a role in its development [11]. Among these factors, intestinal microbiota plays an essential role in transforming primary bile acid into secondary bile acid [12].

Studies have shown that there is a relationship between the imbalance of intestinal microbiota and cholesterol gallstones [12–14]. Keren et al. found that gallstone patients had a decreased microbial diversity, accompanied by a reduction in the beneficial genus *Roseburia* and an enrichment of the uncultivated genus *Oscillospira*, compared with controls [13]. In another study, Wu et al. found significant increases of gut bacterial phylum Proteobacteria and decreases of three gut bacterial genera, *Faecalibacterium*, *Lachnospira*, and *Roseburia* in gallstone patients compared to normal individuals [14]. Wang et al. reported intestinal microbiota imbalance affects bile acid metabolism and is associated with gallstone formation [10].

As an innovative minimally invasive treatment technology, there is no report about the effect of N-GPS on intestinal microbiota for patients with chronic cholecystitis for a long time. To elucidate the effect of N-GPS, this study endeavored to examine the intestinal microbiota and short-chain fatty acids (SCFAs) of patients with benign gallbladder diseases (BG group) at YPO stage (over 1 year postoperative) and at DPR stage (within 1 week preoperative) and healthy controls (healthy control group (HC group)). We employed 16S rDNA Illumina MiSeq sequencing to investigate the intestinal microbial profile and then employed GC-MS technique to analyze SCFAs' levels in fecal of HC group and two stages of BG group. Eventually, this research sought to determine whether N-GPS can lead to beneficial alterations in the intestinal microbiota of patients after performing this surgery for over 1 year.

## 2. Materials and Methods

### 2.1. Study Design and Enrolled Patients

The project was approved by the Ethics Committee of the 909th Hospital (No. 2018-009-01). All experiments were performed in accordance with the Helsinki Declaration and the Rules of Good Clinical Practice. Written informed consent and questionnaires addressing previous and current diseases, lifestyles, and medications were obtained from all participants.

Patients (BG group) diagnosed with gallstone or GPs with ultrasonography and planned for N-GPS were enrolled. Inclusion criteria were as follows: (1) strong echogenic masses visible on ultrasound examination, suggesting gallstone or GP-like changes; (2) good gallbladder contractile function; and (3) clear willingness to preserve the gallbladder. Exclusion criteria included the following: (1) acute cholecystitis, (2) nonfunctional gallbladder, and (3) patients not agreeing to N-GPS.

Healthy controls (HC group) were also recruited. Inclusion criteria were as follows: (1) participating in physical examination and (2) ultrasound examination results suggesting the structure and function of the liver, gallbladder, spleen, and pancreas were normal. Exclusion criteria included the following: (1) ultrasound examination results suggesting disease of gallstone or GPs and (2) having a clear medical history of gastrointestinal tumor, IBD, hypertension, or diabetes.

### 2.2. Collection of Fecal Samples

**Figure 1:**
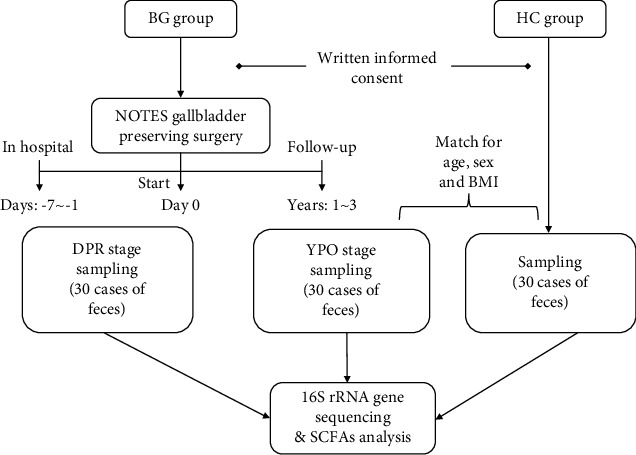
Study design and sample collection timeframes.

Fecal samples from BG group within 1 week before surgery (DPR stage, during 2018–2020) and over 1 year after surgery (YPO stage, during 2022) and HC group (during 2022) whose sex, age, and BMI matched with patients at YPO stage were collected (Figure 1). Stool samples were taken from a deep part of the fresh stool using a sampling spoon and quickly placed in a special stool collection tube. The tubes containing samples were sealed, labeled, and transferred to a −80°C freezer for storage within 2 h.

### 2.3. DNA Extraction and 16S rRNA Gene Sequencing

Total DNA was extracted using the OMEGA Soil DNA Kit, and the extracted DNA was used as the template [15, 16]. The 16S rRNA gene sequences were amplified from each extracted DNA sample using primer pairs 338F (forward primer 5⁣′-ACTCCTACGGGAGGCAGCA-3⁣′) and 806R (reverse primer 5⁣′-GGACTACHVGGGTWTCTAAT-3⁣′) targeting the hypervariable V3–V4 region. DNA purification, 16S rRNA gene amplification, and Illumina MiSeq sequencing were entrusted to Shenzhen Microeco Technology Ltd. (Shenzhen, China). The raw Illumina read data for all samples were deposited under the NCBI BioProject with accession number PRJNA854270.

### 2.4. Processing and Analysis of Sequencing Data

The raw data was performed using QIIME 2 (version 2022.2) [17]. Briefly, raw sequence data were demultiplexed using q2-demux plugin, and primers were cut with q2-cutadapt plugin. Sequences were then quality filtered, denoised, merged, and chimera removed to obtain amplicon sequence variants (ASVs) using the DADA2 plugin (version 1.22.0) [18]. The taxonomy annotation of each ASV representative sequence was analyzed using the Greengenes2 database (version 2022.10) [19]. Firmicutes/Bacteroidota (F/B) in each category was calculated by total Firmicutes absolute accounts in each category divided by total Bacteroidota absolute accounts in each category.

### 2.5. Diversity Analysis of Intestinal Microbiota

To determine the richness of sample species composition, the *α*-diversity indexes (observed ASVs, Shannon) were evaluated by QIIME 2. Statistically significant difference in *α*-diversity indexes between BG group at DPR stage, BG group at YPO stage, and HC group was assessed using the Wilcoxon test. Principal coordinate analysis (PCoA) was performed to identify differences in microbial community composition for three categories by visualizing the samples on the coordinate map [20]. Analysis of similarities (ANOSIM) was used to analyze the statistical significance.

### 2.6. Differential Analysis of Intestinal Microbiota

To detect taxa with significant differences in richness, we used Linear discriminant analysis Effect Size (LEfSe) [21] and Deseq2 methods [22, 23]. LEfSe analysis was applied to a table of features (obtained from QIIME2) to identify potential marker taxa that could be distinguished for three categories. Significantly differential taxa were defined with an LDA score (log10) over 2.0. The Kruskal–Wallis test was applied as a factorial test for the taxa. The level of significance was alpha = 5%.

### 2.7. Network Analysis of Co-Occurrence in Intestinal Microbiota

Co-occurrence networks were constructed using SParCC algorithm through MicrobiomeAnalyst 2.0 server [24]. To explore the interaction between the gut microbiota of three categories, ASVs in 20% samples containing minimum counts 51 were selected for Co-occurrence network analysis. Correlations were calculated using SParCC algorithm with a correlation threshold *r* > 0.3 and *p* value < 0.05. The correlation results were then visualized with the Cytoscape software (v3.10.2) [25].

### 2.8. Differential Analysis of Predicted Metagenomic Function

The Phylogenetic Investigation of Communities by Reconstruction of Unobserved States (PICRUSt2) pipeline was used to predict metagenomic function of intestinal microbiota [26]. LEfSe method was applied to analyze the significant difference for KEGG pathway L3 in the studied categories with a LDA score (log10) over 2.5.

### 2.9. SCFA Analysis of Fecal Samples

To reduce the amount of test samples, fecal samples from five to seven patients with the same pathology (Type 1, gallstone; Type 2, GPs; Type 3, gallstone and GPs) were taken in equal amounts and merged into one specimen. Correspondingly, samples of each category (HC group and DPR and YPO stage of BG group) were merged into five specimens individually. Finally, a total of 15 specimens were obtained for SCFA testing.

For SCFA analysis, stool samples were homogenized for 1 min with 500 *μ*L of water and 100 mg of glass beads and then centrifuged at 4°C for 10 min at 16,000 × *g*. Two hundred microliters of supernatant was extracted with 100 *μ*L of 15% phosphoric acid and 20 *μ*L of 375 *μ*g/mL 4-methylvaleric acid solution as internal standard (IS) and 280 *μ*L ether. Subsequently, the samples were vortexed for 1 min and centrifuged at 4°C for 10 min at 16,000 × *g*, and the supernatant was transferred into the vial prior to GC-MS analysis. The GC analysis was performed on trace 1310 gas chromatograph (Thermo Fisher Scientific, United States). Mass spectrometric detection was performed on ISQLT (Thermo Fisher Scientific, United States).

### 2.10. Statistical Analysis

Statistical analyses were performed using GraphPad Prism (GraphPad Software). Chi-square test was used for comparison of gender composition among multiple categories; one-way ANOVA with Tukey's test (parametric) and Kruskal-Wallis with Dunn's test (nonparametric) were used for multiple comparisons; the Wilcoxon rank-sum test was used for comparisons between two categories. The Pearson correlation was used to evaluate trends between the absolute abundance of genus and the concentration level of SCFA. Unless otherwise specified, bioinformatics analysis was completed using the Wekemo Bioincloud (https://www.bioincloud.tech) [19].

## 3. Results

### 3.1. Characteristics of Participants

The study included 30 patients consisting of 12 females and 18 males, with an average age of 46.5 ± 12.2 and 48.7 ± 12.2 years and an average BMI of 23.9 ± 3.84 and 23.6 ± 4.14 at DPR stage and YPO stage, respectively. The study also contained 30 healthy controls consisting of 11 females and 19 males, with an average age of 47.6 ± 10.6 years and an average BMI of 23.8 ± 3.19 (Table 1). There wasn't statistically difference between the two stages of BG group and HC group in sex, age, and BMI index. Via-gastric pathway contained 25 cases and via-rectal pathway contained five cases of patients who had performed on N-GPS. Of all patients, 13 cases had gallstones and six cases had both gallstones and GPs, while 11 cases had only GPs (Table 1).

### 3.2. Annotation and Comparison of Intestinal Microbiota

**Figure 2:**
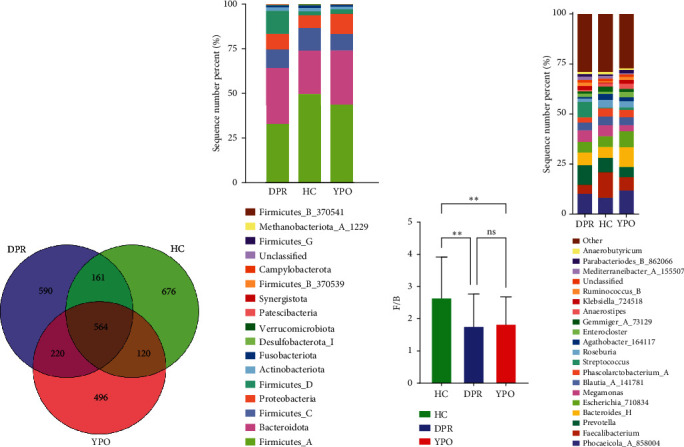
Intestinal microbial composition in two stages of BG group and HC group. (a) Venn diagram of the number of ASVs in HC group and DPR and YPO stage of BG group. (b) Bar plot of microbiota community composition at the phylum level. (c) The ratio of Firmicutes/Bacteroidota, mean ± standard deviation, Tukey's test. (d) Bar plot of microbiota community composition at the genus level.

Illumina MiSeq sequencing of 16S rRNA gene results showed that a total of 4,819,580 raw reads (input sequences before filtering) across all 90 samples were identified, with an average of 53,551 ± 7649 reads per sample (Supporting Information 2: Table S1). After filtering, denoising, merging, and chimera removal, 2,619,883 sequences were generated. The average number of denoised sequences obtained from the HC group, BG_DPR stage, and BG_YPO stage was 31,680 ± 2728, 29,248 ± 3485, and 26,400 ± 2553, respectively (Supporting Information 2: Table S2). The final sequences contained 2827 ASVs; a Venn diagram showed that HC group, BG_DPR stage, and BG_YPO stage had 676, 590, and 496 unshared ASVs, respectively, with 564 shared ASVs by three categories (Figure 2(a)).

There were differences existing in the intestinal microbiota community composition. At the phylum level, in comparison to HC group, both BG at DPR stage and BG at YPO stage depleted Firmicutes_A and enriched Bacteroidota and Proteobacteria; however, in comparison to BG at DPR stage, the depleted of Firmicutes_A was reduced (5.79% vs. 16.69%) and the enriched percentage of Proteobacteria was added (4.74% vs. 1.75%) in BG at YPO stage (Figure 2(b) and Supporting Information 2: Table S3). Compared with HC group, the ratio of Firmicutes to Bacteroidota of BG at DPR stage and BG at YPO stage was both decreased (Figure 2(c)). At the genus level, in comparison to HC group, both BG at DPR stage and BG at YPO stage depleted *Faecalibacterium*, *Roseburia*, and *Agathobacter_164117*, whereas enriched *Phocaeicola_A_858004*, *Bacteroides_H*, and *Enterocloster*; however, in comparison to BG at DPR stage, the depleted percentage of *Faecalibacterium* (6.2% vs. 8.18%), *Roseburia* (0.76% vs. 2.19%), and *Agathobacter_164117* (0.87% vs. 1.99%) was reduced and the enriched percentage of *Phocaeicola_A_858004* (3.61% vs. 1.9%), *Bacteroides_H* (4.38% vs. 1.15%), and *Enterocloster* (1.43% vs. 0.26%) was added in BG at YPO stage (Figure 2(d) and Supporting Information 2: Table S4). It is worth noting that, in comparison to HC group, both BG at DPR stage and BG at YPO stage enriched *Streptococcus*; however, in comparison to BG at DPR stage, the enriched percentage of *Streptococcus* was remarkably reduced (0.63% vs. 7.33%) in BG at YPO stage.

### 3.3. Alpha Diversity of Intestinal Microbiota

**Figure 3:**
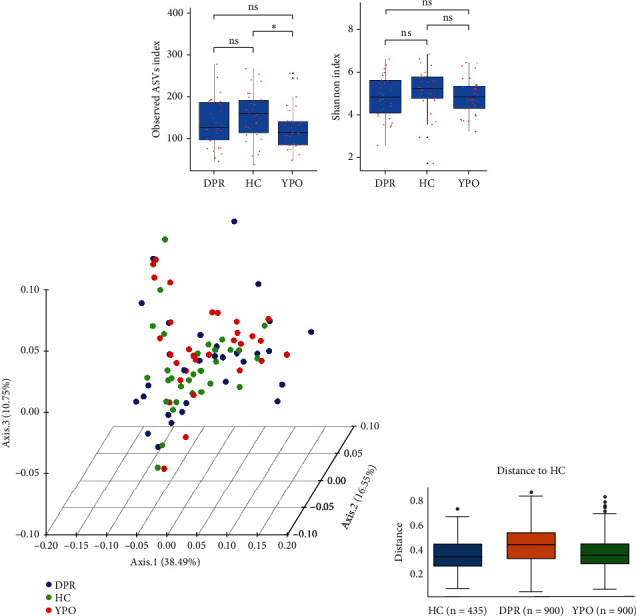
Diversity analysis of intestinal microbiota for two stages of BG group and HC group. (a) Observed ASVs and Wilcoxon Rank Sum test. (b) Shannon index and Wilcoxon Rank Sum test. (c) Principal coordinate analysis (PCoA) based on weighted UniFrac distances. (d) ANOSIM analysis based on weighted UniFrac distances. A statistical significance was defined when *p* < 0.05. Significant levels: ⁣^∗^*p* < 0.05, ⁣^∗∗^*p* < 0.01, and ⁣^∗∗∗^*p* < 0.001. ns: no significance.

The *α*-diversity of the bacterial community were expressed as richness (observed ASVs) and diversity (Shannon index) in the BG group at DPR stage, BG group at YPO stage, and HC group (Figure 3). Comparing with HC group, only BG group at YPO stage showed a significant difference in observed ASVs, suggesting the richness of gut microbiota in the BG group at YPO stage was decreased (Figure 3(a)); no significant difference was observed at both stages of BG group in Shannon index, suggesting that the diversity of gut microbiota did not change significantly (Figure 3(b)).

### 3.4. Beta Diversity of Intestinal Microbiota

The intestinal microbiota of HC group, BG_DPR stage, and BG_YPO stage were not completely coincident in the PCoA diagram based on the weighted UniFrac distance, with the first two main coordinates having the largest contribution rate of 38.49% and 16.55%, respectively (Figure 3(c)). ANOSIM analysis of beta diversity among HC group and two stages of BG group was conducted based on the weighted UniFrac distance, too. It was found that the difference between BG_DPR stage and HC group was statistically significant (*Q* < 0.01; *Q* value is the corrected *p* value), whereas the difference between BG_YPO stage and HC group wasn't statistically significant (*Q* > 0.05) (Figure 3(d) and Supporting Information 2: Table S5).

### 3.5. Differential Analysis of Intestinal Microbiota

**Figure 4:**
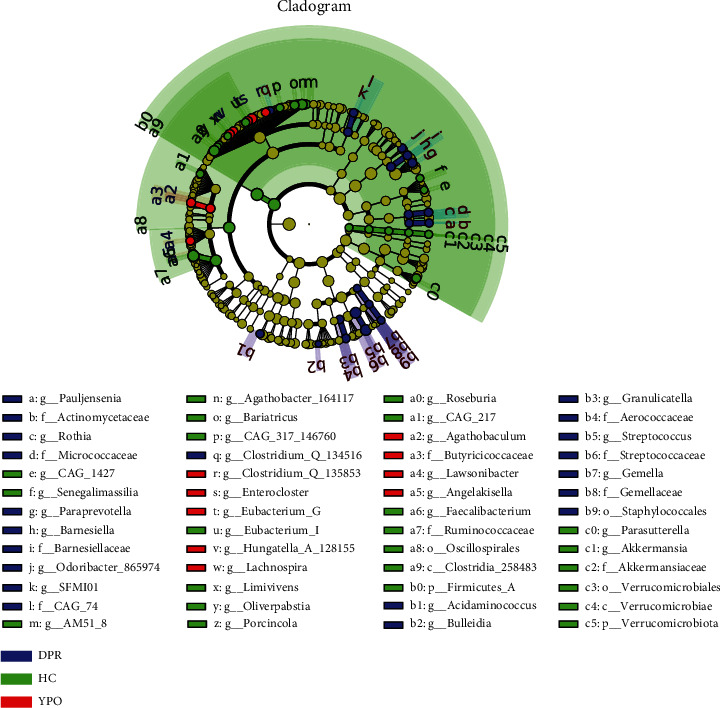
Differential analysis of intestinal microbiota with LEfSe cladogram for two stages of BG group and HC group. Microbial taxa with linear discriminant analysis (LDA) score greater than 2.0 were presented. BG_DPR stage (blue), HC group (green), and BG_YPO stage (red). *p* value < 0.05.

The application of LEfSe can help find good biomarkers. The LEfSe comparison suggested the enrichment of some specific taxa among the microbiota of HC group and two stages of BG group. The microbiota of HC group enriched unique genus, such as *Faecalibacterium*, *Roseburia*, *Agathobacter_164117*, *Parasutterella*, *Bariatricus*, *Eubacterium_I*, *Oliverpabstia*, and *Akkermansia*, most of which belonged to Class Clostridia_258483 (Phylum Firmicutes_A) and Verrucomicrobiae (Phylum Verrucomicrobiota). The microbiota of BG_DPR stage enriched unique taxa, such as *Streptococcus*, *Paraprevotella*, *Barnesiella*, *Odoribacter_865974*, *Rothia*, and *Granulicatella*, most of which belonged to Class Bacilli (Phylum Firmicutes_D), Actinobacteria (Phylum Actinobacteriota), and Bacteroidia (Phylum Bacteroidota), whereas the microbiota at BG_YPO stage enriched unique taxa, such as *Enterocloster*, *Lachnospira*, *Eubacterium_G*, and *Agathobaculum*, most of which belonged to Class Clostridia_258483 (Phylum Firmicutes_A) (Figure 4).

**Figure 5:**
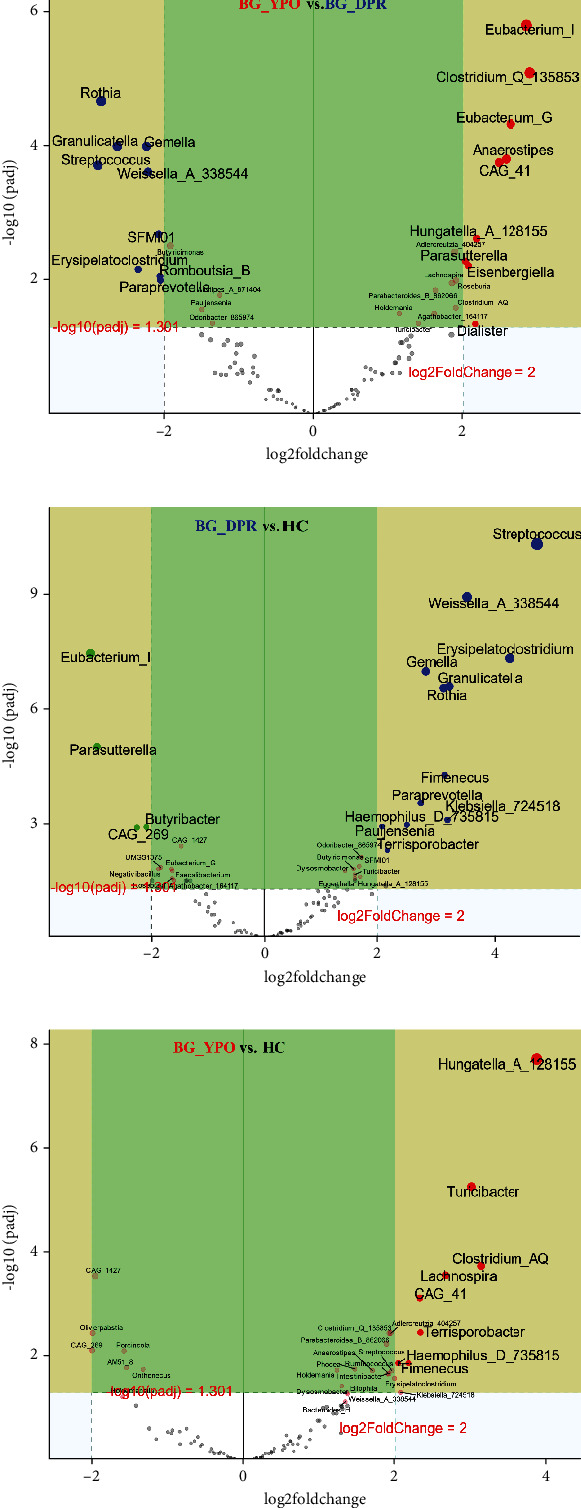
Differential analysis of intestinal microbiota with Deseq2 volcano plot for two stages of BG group and HC group. Volcano plot show identified key gut microbes by Deseq2 analysis that (a) significantly altered between BG_DPR stage and BG_YPO stage, (b) significantly altered between BG_DPR stage and HC group, and (c) significantly altered between BG_YPO stage and HC group. A *Q* value of < 0.05 and fold change of > 2 were set as the thresholds for significant differential taxa.

Deseq2 methods were employed to further identify key intestinal microbiota that significantly altered between two kinds of samples, as shown in volcano plot (*Q* < 0.01, |log2 fold change| > 2) with the top 50 base mean absolute accounts of microbiota. With BG at DPR stage as control, 18% (nine genera) intestinal microbiota significantly increased, including *Eubacterium_I*, *Clostridium_Q_135853*, *Eubacterium_G*, *Anaerostipes*, *CAG_41*, *Hungatella_A_128155*, *Adlercreutzia_404257*, *Parasutterella*, and *Eisenbergiella*, and 18% (nine genera) intestinal microbiota significantly decreased, including *Rothia*, *Gemella*, *Granulicatella*, *Streptococcus*, *Weissella_A_338544*, *SFMI01*, *Erysipelatoclostridium*, *Romboutsia_B*, and *Paraprevotella* in BG at YPO stage (Figure 5(a)). With HC group as control, 24% (12 genera) intestinal microbiota significantly increased, including *Streptococcus*, *Weissella_A_338544*, *Erysipelatoclostridium*, *Gemella*, *Granulicatella*, *Rothia*, *Fimenecus*, *Paraprevotella*, *Klebsiella_724518*, *Haemophilus_D_735815*, *Pauljensenia*, and *Terrisporobacter*, and 8% (four genera) intestinal microbiota significantly decreased, including *Eubacterium_I*, *Parasutterella*, *Butyribacter*, and *CAG_269* in BG at DPR stage (Figure 5(b)). Similarly, with HC group as control, 16% (eight genera) intestinal microbiota significantly increased in BG at YPO stage, including *Hungatella_A_128155*, *Clostridium_AQ*, *Turicibacter*, *Lachnospira*, *Terrisporobacter*, *CAG_41*, *Fimenecus*, and *Haemophilus_D_735815* (Figure 5(c)).

### 3.6. Network Analysis of Intestinal Microbiota

**Figure 6:**
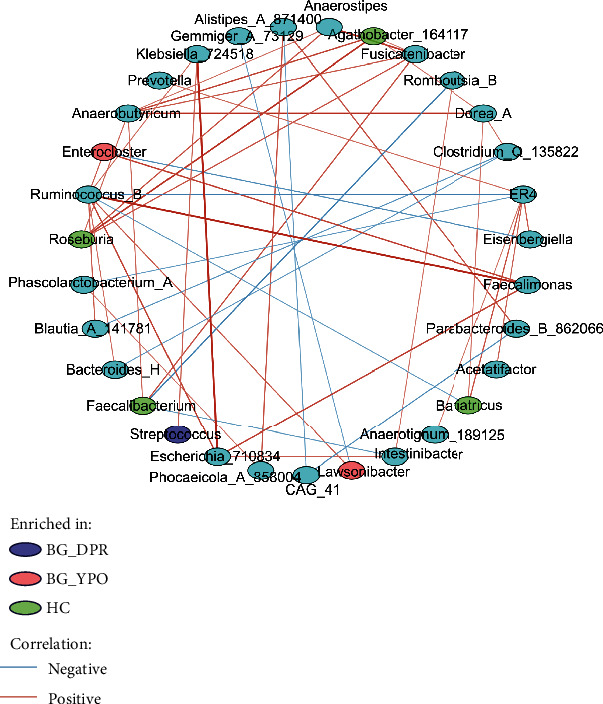
Correlation network analysis of total intestinal microbiota at genus level. ASVs in 20% samples containing minimum counts 51 were selected; correlations were calculated using SparCC algorithm with a correlation threshold *r* > 0.3 and *p* value < 0.05. The center of the image is the correlation network, with nodes representing taxa at the genus level and edges representing correlations between taxa pairs. The red and blue lines represent positive and negative correlations, respectively, and the darker line color and thicker line indicate the greater absolute value of the correlation coefficient.

As characteristic differential taxa find in LEfSe, the change of abundance of these taxa may have profound effect on intestinal microbiota. *Streptococcus*, enriched in BG group at DPR stage, was positively correlated with *Klebsiella_724518* (*r* = 0.3643). *Faecalibacterium*, enriched in HC group, was positively correlated with *Fusicatenibacter* (*r* = 0.4269) and negatively correlated with *Intestinibacter* (*r* = −0.3169) and *Romboutsia_B* (*r* = −0.4211). *Enterocloster*, enriched in BG group at YPO stage, was negatively correlated with *Eisenbergiella* (*r* = −0.3591) and positively correlated with *Faecalimonas* (*r* = 0.4747) and *Ruminococcus_B* (*r* = 0.3766) (Figure 6, Supporting Information 1: Figure S2, and Supporting Information 2: Table S6).

As high abundance level genus may play major roles in gut microbiota, *Ruminococcus_B* (Figure 6, Supporting Information 1: Figure S2, and Supporting Information 2: Table S7) was positively correlated with *Bacteroides_H* (*r* = 0.3308), *Blautia_A_141781* (*r* = 0.3909), *Enterocloster* (*r* = 0.3766), *Escherichia_710834* (*r* = 0.4703), *Faecalimonas* (*r* = 0.6367), *Klebsiella_724518* (*r* = 0.3308), and *Lawsonibacter* (*r* = 0.3927) and negatively correlated with *Bariatricus* (*r* = −0.3071) and *ER4* (*r* = −0.4265) (Figure 6, Supporting Information 1: Figure S2, and Supporting Information 2: Table S6).

### 3.7. Differential Analysis of Predicted Metagenomic Function

**Figure 7:**
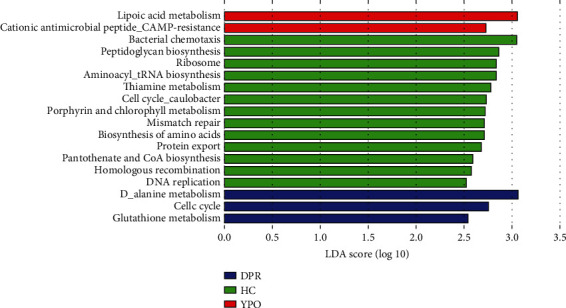
Differences in KEGG pathway L3 level of metabolic pathways of two stages of BG group and HC group predicted by PICRUSt2. Corresponding metagenomic functions for metabolic pathways with 16S rDNA sequencing data of gut microbiota in two stages of BG group and HC group were obtained through PICRUSt2; statistical analysis was done by LEfSe soft (LDA threshold 2.50).

Corresponding metagenomic function with 16S rDNA sequencing data was predicted by PICRUSt2, difference analysis for predicted metagenomic function of KEGG pathway L3 was performed by LEfSe with LDA threshold 2.5, and the results can be seen from Figure 7.

In BG group at DPR stage, D-alanine metabolism, glutathione metabolism, and cell cycle were enriched; in BG group at YPO stage, lipoic acid metabolism and cationic antimicrobial peptide (CAMP) resistance were enriched, whereas in HC group, aminoacyl-tRNA biosynthesis, bacterial chemotaxis, DNA replication, porphyrin, and chlorophyll metabolism were enriched (Figure 7).

### 3.8. SCFA Analysis of Fecal Samples

**Figure 8:**
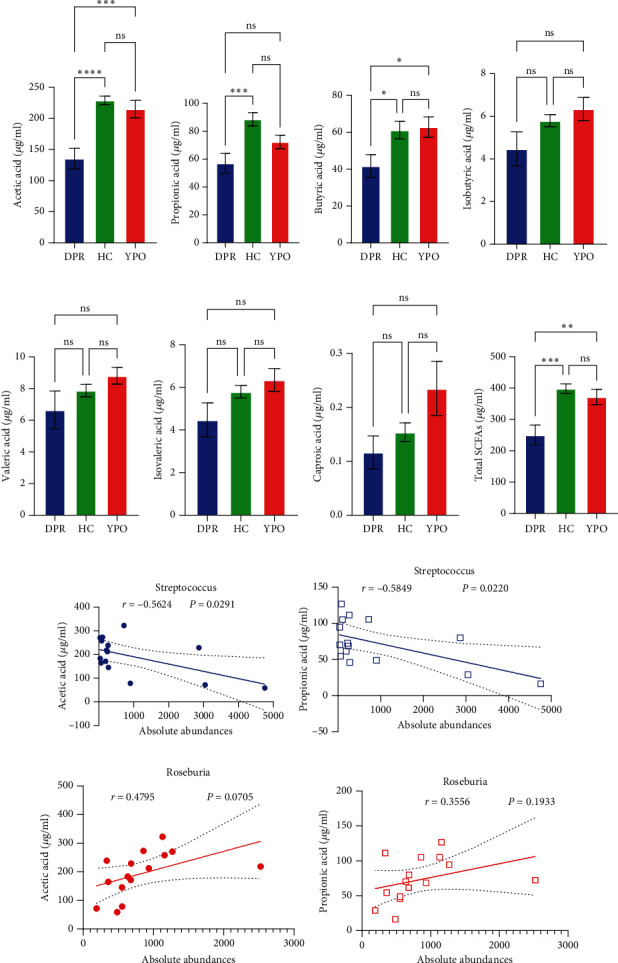
Relationships between SCFA concentration of fecal and enriched/deleted taxa in gut microbiota of two stages of BG group and HC group. (a–h) Comparison for SCFA concentration among HC group and two stages of BG group. Data are expressed as the mean ± standard deviation. The statistic difference of various indicators was determined by one-way ANOVA (Tukey's test). Significant levels: ⁣^∗^*p* < 0.05, ⁣^∗∗^*p* < 0.01, ⁣^∗∗∗^*p* < 0.001, and ⁣^∗∗∗∗^*p* < 0.0001. ns: no significance. (i–l) The relationship of main enriched genera and SCFA level.

Compared with DPR stage of BG group, both HC group and YPO stage of BG group increased concentrations of acetic acid (*p*_HC⁣vs.DPR_ < 0.0001 and *p*_*YPO* vs.DPR_ < 0.001, Figure 8(a)), butyric acid (*p*_HC⁣vs.DPR_ < 0.05 and *p*_*YPO* vs.DPR_ < 0.05, Figure 8(c)), and total SCFAs (*p*_HC⁣vs.DPR_ < 0.01 and *p*_*YPO* vs.DPR_ < 0.01, Figure 8(h)); in addition, HC group increased concentrations of propionic acid (*p*_HC⁣vs.DPR_ < 0.001, Figure 8(b)), while compared with HC group, YPO stage of BG group has no difference for acetic acid (*p*_YPO⁣vs.HC_ > 0.05, Figure 8(a)), propionic acid (*p*_YPO⁣vs.HC_ > 0.05, Figure 8(b)), butyric acid (*p*_YPO⁣vs.HC_ > 0.05, Figure 8(c)), and total SCFAs (*p* > 0.05, Figure 8(h)). There is no difference for isobutyric acid (Figure 8(d)), valeric acid (Figure 8(e)), isovaleric acid (Figure 8(f)), and caproic acid (Figure 8(g)) among two stages of BG group and HC group statistically.

Pearson's coefficient (*r*) and significance (*p*) were calculated separately for each SCFA (Supporting Information 2: Table S8) and main enriched genus (Supporting Information 2: Table S9) in three categories. There was a negative correlation between *Streptococcus* and fecal acetic acid (*p* = 0.0291) (Figure 8(i))/propionic acid (*p* = 0.0220) (Figure 8(j)), whereas there was a positive correlation between *Roseburia* and fecal acetic acid (*p* = 0.0705) (Figure 8(k))/propionic acid (*p* = 0.1933) (Figure 8(l)).

## 4. Discussion

Chronic cholecystitis is generally caused by chronic inflammation of the gallbladder from long-term existing gallbladder stones, or the repeated and prolonged onset of acute cholecystitis. Its clinical manifestations vary greatly and can manifest as asymptomatic, recurrent discomfort or abdominal pain in the upper right abdomen, or acute attacks. The typical abdominal ultrasound examination shows thickening and roughness of the gallbladder wall (wall thickness ≥ 3 mm), and the presence of gallstones can manifest as strong echoes and posterior echogenicity in the gallbladder. According to the presence of stones in the gallbladder, it can be divided into calculous cholecystitis and noncalculous cholecystitis [27]. For patients with asymptomatic gallstones, regular follow-up examination is recommended rather than cholecystectomy [28]. Whereas LC is typically recommended for chronic cholecystitis [4], N-GPS is the application of NOTES technology for removing gallstones and GPs while preserving the gallbladder, offering a potential alternative to LC for chronic cholecystitis (Supporting Information 1: Figure S1). Understanding changes in the gut microbiota will provide us with a new perspective on the impact of N-GPS.

Wang et al. reported that intestinal microbiota imbalance affects bile acid metabolism and is associated with gallstone formation [10]. They recruited 30 patients and 30 healthy individuals in the gallstone group and control group, respectively; the differences in age, sex, and BMI index were not statistically significant. The subjects in these two groups had not taken antibiotics for 3 months and did not have metabolic diseases. The patients in the gallstone group were confirmed to be free of bile duct stones and GPs. At the phylum level, the number of Firmicutes was significantly reduced in the gallstone group, and the F/B ratio was also significantly decreased. At the genus level, the result suggested *Ruminococcus gnavus* could be used as a biomarker in the gallstone group, while *Prevotella* 9 and *Faecalibacterium* could be used as biomarkers in the control group [10].

In our previous study, we investigated the alteration in the intestinal microbiota of patients between preoperative stages DPR stage (1–7 days presurgery) and different postoperative stages DPO stage (1–7 days postsurgery) [29], MPO stage (1–3 months postsurgery) [30], and YPO stage (1–3 years postsurgery, data not published). We find that there was no significant difference in alpha diversity at YPO stage with DPR stage as the disease control group, which suggests YPO stage is a relative stabilized period for showing the effect of alteration of gut microbiota (data not published). In this study, we further recruited the same number of volunteers as the HC group, matching sex, age, and BMI indexes with BG group at YPO stage, to elucidate whether the gut microbiota of patients at YPO stage of surgery restored and closed to those of HC group.

In this paper, we find that at the phylum level, compared with HC group, both stages of BG group enriched Bacteroidota; the ratio of Firmicutes to Bacteroidota was both decreased. However, the difference was that BG at DPR stage enriched Firmicutes_D and depleted more Firmicutes_A, whereas BG at YPO stage enriched Proteobacteria and depleted less Firmicutes_A. At the genus level, compared with HC group, both stages of BG group depleted *Faecalibacterium*, *Phascolarctobacterium_A*, *Roseburia*, and *Agathobacter_164117* and enriched *Phocaeicola_A_858004*, *Bacteroides_H*, and *Enterocloster.* However, BG at YPO stage reduced the difference with HC group in SCFA-producing bacteria (*Phascolarctobacterium_A*, *Roseburia*, and *Agathobacter_164117*) and pathobionts (*Streptococcus*) but added the bacteria related to bile acid metabolism (*Phocaeicola_A_858004*, *Bacteroides_H*, and *Enterocloster*).

The result of alpha diversity of Shannon index showed that the difference between the two stages of BG group and HC group wasn't statistically significant, while the result of beta diversity based on the weighted UniFrac distance suggested that the structure of intestinal microbiota of BG group at YPO stage was closer to HC group. The comparative methods, such as LEfSe and Deseq2, suggested that some specific taxa presented among the microbiota of HC group and two stages of BG group were worthy of attention. The microbiota of HC group enriched unique genera, such as *Faecalibacterium* and *Roseburia*; the microbiota of BG_DPR stage enriched unique genera, such as *Streptococcus*, *Odoribacter_865974*, and *Rothia*, whereas the microbiota at BG_YPO stage enriched unique genera, such as *Enterocloster* (related with bile acid metabolism), *Hungatella_A_128155* (related with bile acid metabolism), and *Lachnospira*. Network analysis of intestinal microbiota suggests that high abundance level genus *Ruminococcus_B* enriched in HC group was related with changes of many genera.

In BG_DPR category, D-alanine metabolism, glutathione metabolism, and cell cycle were enriched, which suggested that the synthesis of bacterial cell wall was increased, and bacterial reproduction was accelerated; therefore, enriched pathobionts and predicted function at BG_DPR stage may be related with chronic cholecystitis. In BG_YPO category, lipoic acid metabolism and CAMP resistance were enriched, which suggested that the coenzyme with strong antioxidant and detoxifying properties and the capacity for protecting against bacterial infection were added.

There was a significant negative correlation between *Streptococcus* and fecal acetic acid/propionic acid, whereas there was a positive correlation between *Roseburia* and fecal acetic acid/propionic acid.

A reasonable assumption is N-GPS improved the intestinal hepatic circulation of bile by enriching genus, such as *Phocaeicola_A_858004*, *Bacteroides_H*, and *Enterocloster*; then, the improved gut environment of pH probably promoted the proliferation of SCFA-producing bacteria such as *Roseburia*; and finally, the increased SCFA concentration level inhibited the growth of pathogenic bacteria.

There were some limits in this paper. First, as this was a real-world study, we did not exclude patients who used antibiotics before surgery; the preoperative gut microbiota of patients might be partially affected by antibiotics. If possible, recruiting patients without antibiotics before surgery as a positive control was a more ideal choice. Second, in our paper, we recruited patients with gallstones and GPs with a ratio nearly 1:1, which might decrease the effect of gut microbiota of patients with gallstones in the BG group. There were few literatures exploring the imbalance of gut microbiota in patients with GPs; therefore, we did not know the dysbiosis of gut microbiota in these participants. With more surgery cases to be obtained, we need to differentiate the patients with gallstone and GPs. Third, as an innovative surgery, the sample size was relatively small, and the study was a single center. With the accumulation of cases for N-GPS, the above deficiency may be resolved in the future.

## 5. Conclusion

This study compared the intestinal microbiota of patients with benign lesions of the gallbladder and chronic cholecystitis at preoperative stage (DPR stage) and at postoperative stage (YPO stage) with HC group. After more than 1 year of surgery, patients can reduce harmful pathogens by increasing intestinal SCFA-producing bacteria, such as *Faecalibacterium* and *Roseburia*, and increasing the concentration of nonbranched SCFAs (acetic acid, propionic acid, and butyric acid). Predicting the function of metagenomes can resist to lipid metabolism disorders and enhance immune regulatory ability, too. Research had shown that N-GPS is an effective method for treating benign gallbladder lesions and chronic cholecystitis. It improved the imbalance of the gut microbiota and gradually brought the postoperative gut microbiota of patients closer to that of the healthy population. Furthermore, taking probiotics for increasing *Faecalibacterium* and *Roseburia* is probably beneficial for preventing the recurrence of cholecystitis after N-GPS.

## Figures and Tables

**Table 1:** Baseline data of two stages of BG group and HC group.

**Groups**	**Surgical approach**	**Type of lesion**	**Sex**	**Categories**	**Age**	**BMI**
HC group			Female (11); male (19)	HC group	47.6 ± 10.6	23.8 ± 3.19
BG group	Stomach (25); rectum (5)	Stone (13); polyps (11); polyps + stone (6)	Female (12); male (18)	BG_DPR	46.5 ± 12.2	23.9 ± 3.84
				BG_YPO	48.7 ± 12.2	23.6 ± 4.14

## Data Availability

The DNA sequencing data in this article is deposited in the NCBI BioProject database (https://www.ncbi.nlm.nih.gov/bioproject), with the accession number PRJNA854270.

## Supporting Information

Supporting Information 1 Figure S1. A case of NOTES gallbladder-preserving surgery (N-GPS) by transrectal approach. (a) The intestinal cavity was cleaned thoroughly with the endoscope. (b) A dual knife was used to cut through the intestinal wall. (c) The gallbladder was found at the lower edge of the liver. (d) The gallbladder was cut open with a dual knife. (e) A mesh bag was used to remove the stone. (f) The gallbladder incision was clamped with four hemostatic clips. (g) The intestinal wall incision was tightly closed with six hemostatic clips. (h) The removed gallstone was shown. Figure S2. Enriched genera in two stages of BG group and HC group. (a) *Streptococcus* enriched in BG_DPR. (b) *Faecalibacterium* enriched in HC. (c) *Enterocloster* enriched in BG_YPO. (d) *Ruminococcus_B* enriched in HC.

Supporting Information 2 Table S1. Demultiplexed sequence counts of total intestinal microbiota. Table S2. Statistical table of the process of generating ASV by DADA2 denoising. Table S3. The relative abundance of top 20 phylum in each category. Table S4. The relative abundance of top 20 genus in each category. Table S5. ANOSIM analysis of beta diversity based on the weighted UniFrac distance. Table S6. Edge table of correlation network using SparCC algorithm for total intestinal microbiota at genus level. Table S7. Node table of correlation network using SparCC algorithm for total intestinal microbiota at genus level. Table S8. SCFA concentrations in 15 merged samples. Table S9. Absolute abundances of top 20 enriched genera calculated in 15 merged samples for SCFA testing.
