# Supplementary material for: Beneficial Alterations of Intestinal Microbiota in Chronic Cholecystitis Patients Treated With NOTES Gallbladder-Preserving Surgery
Source: Gastroenterol Res Pract. 2024 Nov 7;2024:9327118. doi: 10.1155/2024/9327118 (PMC11563709; doi:10.1155/2024/9327118)
Supplement: Supporting Information 1 — Figure S1. A case of NOTES gallbladder-preserving surgery (N-GPS) by transrectal approach. (a) The intestinal cavity was cleaned thoroughly with the endoscope. (b) A dual knife was used to cut through the intestinal wall. (c) The gallbladder was found at the lower edge of the liver. (d) The gallbladder was cut open with a dual knife. (e) A mesh bag was used to remove the stone. (f) The gallbladder incision was clamped with four hemostatic clips. (g) The intestinal wall incision was tightly closed with six hemostatic clips. (h) The removed gallstone was shown. Figure S2. Enriched genera in two stages of BG group and HC group. (a) Streptococcus enriched in BG_DPR. (b) Faecalibacterium enriched in HC. (c) Enterocloster enriched in BG_YPO. (d) Ruminococcus_B enriched in HC. [file 9327118.f1.pptx]

## Slide 1
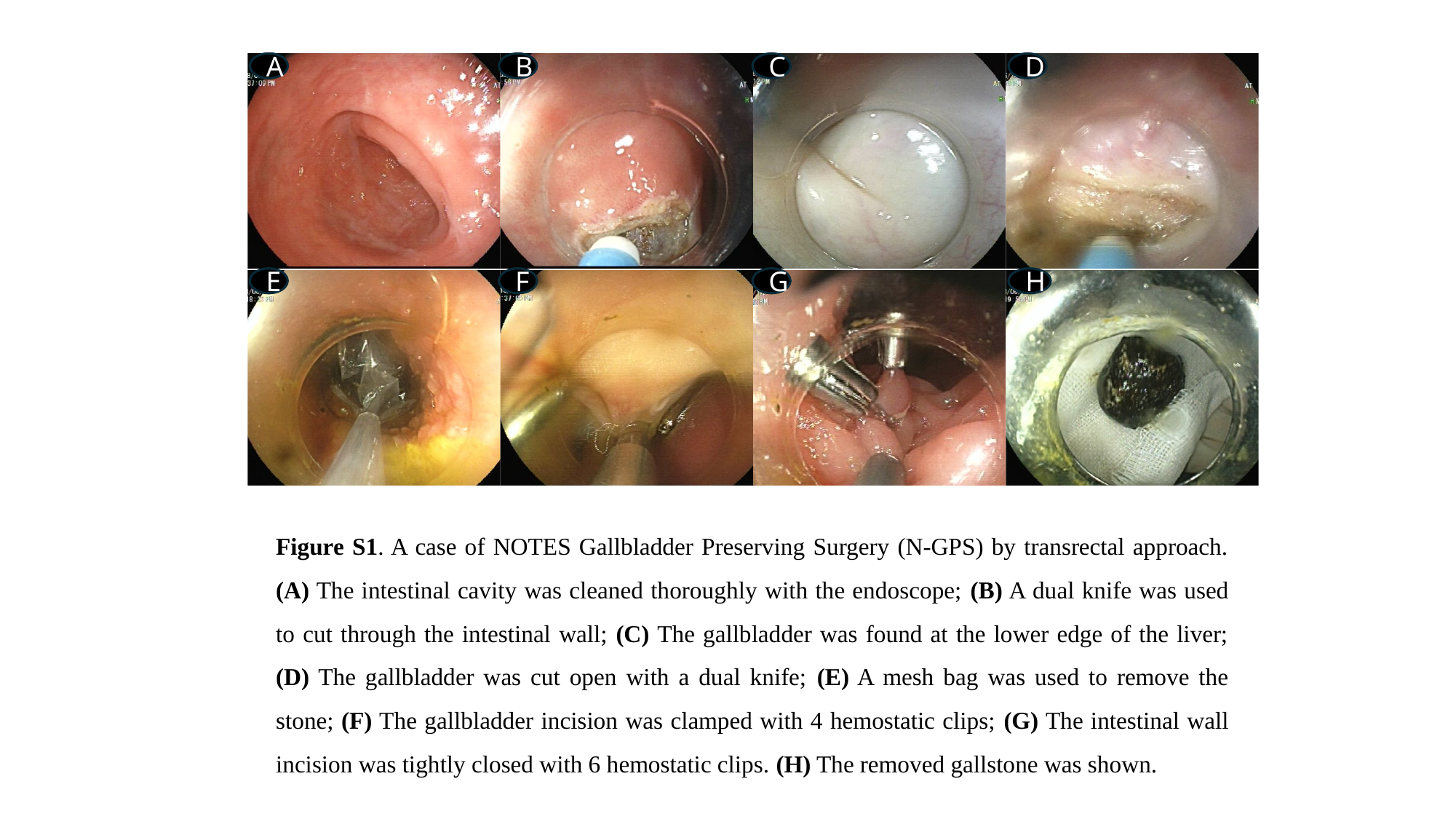

A
B
C
D
E
F
G
H
Figure S1. A case of NOTES Gallbladder Preserving Surgery (N-GPS) by transrectal approach. (A) The intestinal cavity was cleaned thoroughly with the endoscope; (B) A dual knife was used to cut through the intestinal wall; (C) The gallbladder was found at the lower edge of the liver; (D) The gallbladder was cut open with a dual knife; (E) A mesh bag was used to remove the stone; (F) The gallbladder incision was clamped with 4 hemostatic clips; (G) The intestinal wall incision was tightly closed with 6 hemostatic clips. (H) The removed gallstone was shown.

## Slide 2
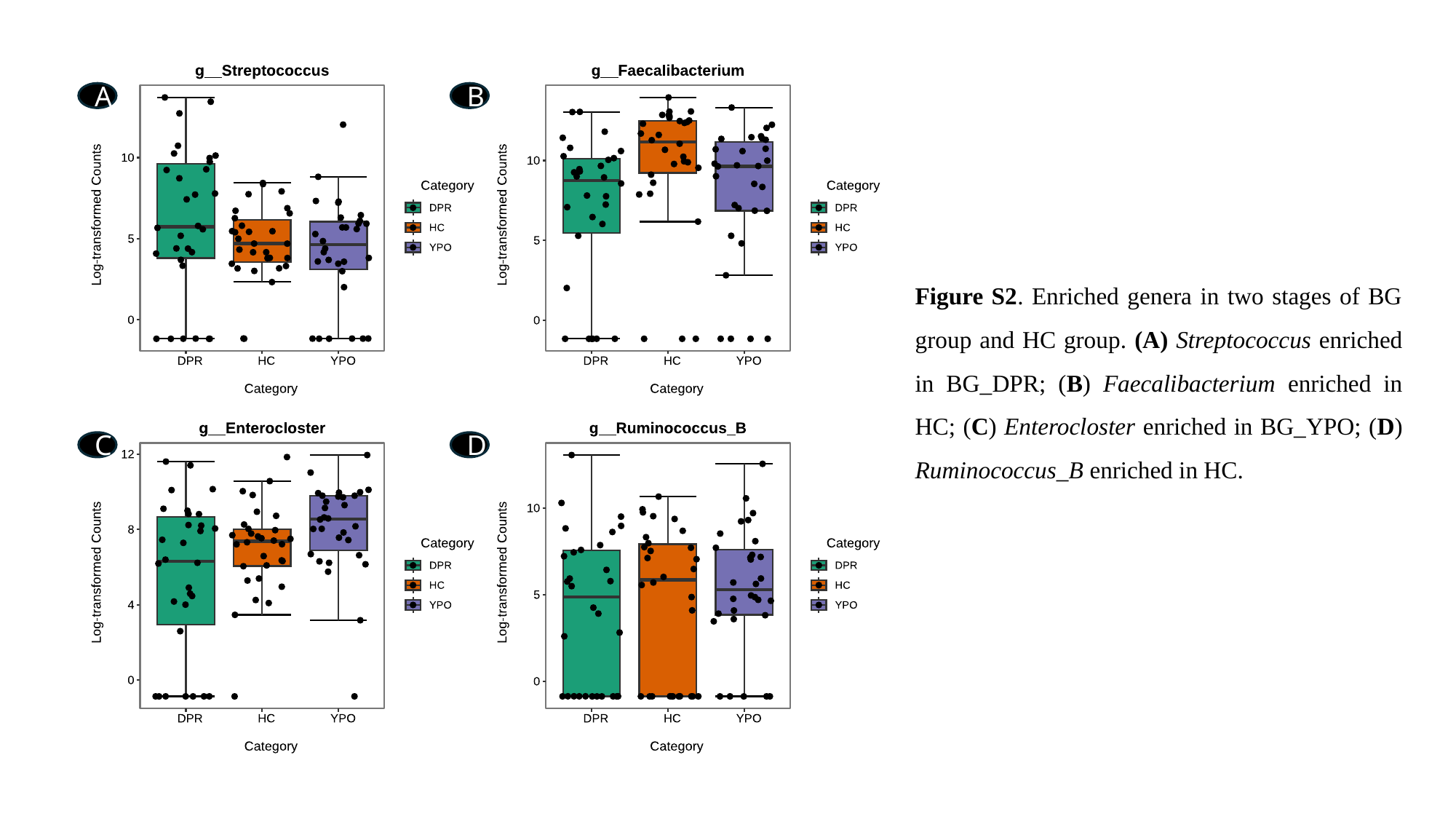

A
B
D
C
Figure S2. Enriched genera in two stages of BG group and HC group. (A) Streptococcus enriched in BG_DPR; (B) Faecalibacterium enriched in HC; (C) Enterocloster enriched in BG_YPO; (D) Ruminococcus_B enriched in HC.
